# Formula of Chloroform

**Published:** 1855-11

**Authors:** 


FORMULA OF CHLOROFORM.—M. Dorvault states that chloroform is easily soluble in water, one part by weight to the 100. When more considerable quantities are required, the Commission of the Society of Pharmacy recommends the following formula: Chloroform 2 to 4 parts, sugar in lumps 12, gum Arabic 5 to 10, and water 100 parts. Pour the chloroform on the sugar placed in a mortar; add next the gum, and then gradually the water, rubbing well. M. Stanislaus Martin suggests the following as preferable: Chlorof. 2 to 6 gram., 1 yolk of egg, 30 gram. of syrup, and 150 of water. Mix the egg with the water, and strain, and then add the syrup and chloroform. The egg is more promptly deposited in proportion to the quantity of chloroform used, but carries no chloroform down, and on shaking the mixture becomes uniform.—(*Bull de Thérap.*) *Ibid.*

